# The impact of growth differentiation factor 15 on the risk of cardiovascular diseases: two-sample Mendelian randomization study

**DOI:** 10.1186/s12872-020-01744-2

**Published:** 2020-10-28

**Authors:** Zhuo Wang, Fangkun Yang, Menghuai Ma, Qinyi Bao, Jinlian Shen, Feiming Ye, Xiaojie Xie

**Affiliations:** Department of Cardiology, Second Affiliated Hospital, Zhejiang University School of Medicine, 88 Jiefang Road, Hangzhou, 310009 Zhejiang China

**Keywords:** Cardiovascular diseases, Growth differentiation factor 15, Mendelian randomization, Atrial fibrillation, Cardioembolic stroke, Coronary artery disease, Myocardial infarction

## Abstract

**Background:**

Growth differentiation factor 15 (GDF-15), a stress responsive cytokine, belongs to transforming growth factor β cytokine superfamily. Some evidence support that it’s involved in inflammation, coagulation, oxidative stress, endothelial dysfunction, and hemostasis. However, it’s still controversial whether GDF-15 directly contributes to the morbidity and mortality of patients suffered with cardiovascular disease (CVD). Besides prospective cohort study and randomized controlled trial, Mendelian randomization (MR) is a genetic epidemiological method that exploits genetic variants as unbiased proxies for modifiable to determine the causal relationships between exposures and health outcomes. Herein, we introduced a two-sample MR approach to evaluate the causal relationships of circulating GDF-15 levels with major CVDs incidence.

**Methods:**

Genetic instruments and summary statistics for two-sample MR analysis were obtained from 5 independent large genome-wide association studies (GWAS) to investigate the causal correlation between circulating GDF-15 levels and 9 CVDs, respectively. Conventional inverse variance weighted method was adopted to evaluate the causality of GDF-15 with different outcomes; weighted median and MR egger were used for sensitivity analyses.

**Results:**

Among 9 SNPs identified from 5 GWASs in 2.6 million individuals, 5 SNPs (rs1227731, rs3195944, rs17725099, rs888663, rs749451) coming from chromosome 19 and containing the PGPEP1 and GDF-15 genes were employed. Based on the instruments, circulating GDF-15 levels significantly linked to the increased risk of cardioembolic stroke, atrial fibrillation, coronary artery disease and myocardial infarction. However, no significant causal association was observed for circulating GDF-15 levels with the incidence of any ischemic stroke, large-artery atherosclerotic stroke, small vessel stroke, heart failure and nonischemic cardiomyopathy.

**Conclusions:**

The MR study provides with genetic evidence for the causal relationship of circulating GDF-15 levels with the increased risk of cardioembolic stroke, atrial fibrillation, coronary artery disease and myocardial infarction, but not any ischemic stroke, large-artery atherosclerotic stroke, small vessel stroke, heart failure and nonischemic cardiomyopathy. It indicates that GDF-15 might be a promising biomarker or potential therapeutic target for some CVDs.

## Introduction

Together with the economic growth and lifestyle change, the incidence of cardiovascular disease is also increasing in the global countries. Cardiovascular disease (CVD) has become an overwhelming cause of morbidity and mortality of population health, which contribute to the current public health concern. Subsequently, the increasing medical insurance costs become an important social economic burden. It’s crucial and significant to figure out the causal elements and therapeutic targets to reduce CVD morbidity and mortality, improve life quality and alleviate medical insurance burden.

Recent researches have demonstrated that metformin, a classical and first-line glucose-lowering drug, is beneficial to cardiovascular outcomes and reduction of total mortality independent of glycemic control. Many different mechanisms beyond glycemic control have been implicated in cardiovascular protection induced by metformin, such as improvements of inflammation, coagulation, oxidative stress, endothelial dysfunction, and hemostasis [1]. Growth differentiation factor-15 (GDF-15) and its receptor GDNF family receptor α-like (GFRAL) are potential molecular targets of metformin, involving in macrophage activation and differentiation of cardiovascular inflammation [2, 3]. GDF-15 is a divergent member of the transforming growth factor-β (TGF-β) superfamily originally identified as macrophage inhibitory cytokine-1 (MIC-1) based on increased mRNA expression associated with macrophage activation. It is also known as placental transformation growth factor (PTGF-β), prostate derived factor (PDF), placental bone morphogenetic protein (PLAB), NSAID activated gene-1 (NAG-1), and PL74. GDF-15 promoter, a locus on chromosome 19, contains p53-transcription factor binding sites that are required and sufficient for the induction of GDF-15 mRNA expression [4]. The major function of this protein is still uncertain, but it has been suggested to have a number of different roles including growth inhibition and induction of apoptosis in epithelial and other tumor cell lines. GDF-15, also known as a stress-responsive protein, is involved in oxidative stress and tissue hypoxia, which might be an effective predictor and promising therapeutic targets for CVD.

The PARADIGM-HF trial has shown that GDF-15 plays independently a prognostic value in patients with heart failure with reduced ejection fraction (HFrEF) in which higher baseline and incremental GDF-15 levels were significantly relevant to mortality and all cardiovascular events, even after adjusting circulating concentrations of N-terminal pro-B-type natriuretic peptide (NT-proBNP) and high-sensitivity cardiac troponin T (cTnT) [5]. Similar results were observed in patient with acute coronary syndrome (ACS) [6], stable coronary artery disease (CAD) [7], and high risk population [8]. Inconsistently, BIOSTAT-CHF trial has reported that GDF-15 levels were not influenced by the presence of atrial fibrillation (AF) in patients with heart failure (HF) [9]. Since those observational studies inevitably blend with some confounding factors (where some factors associated with GDF-15 actually result in the disease) and reverse causality bias (where some patients with CVD may be more likely to higher GDF-15), it remains controversial whether GDF-15 is causally associated with the incidence of CVD.

Mendelian randomization (MR) is an epidemiological robust tool by using genetic variants as proxies for the exposure to infer causality between risk factors and outcome or disease [10]. Because of inherited variants are independent of potentially confounding environmental exposures, the MR method can be a complement for observational trials. Large genome-wide association study (GWAS) has been widely used over the last decade, allowing the conduct of MR analysis without the need to recruit new patients or design additional studies. There was a two-sample MR study based on GWAS that indicated GDF-15 levels uncorrelated to risk of cardiometabolic outcomes [11], including body mass index, waist-hip ratio, waist circumference, whole-body lean mass, fat percentage, fasting glucose, glycated hemoglobin, fasting insulin, low density lipoprotein (LDL)-cholesterol, low density lipoprotein (HDL)-cholesterol, total cholesterol, triglycerides, type 2 diabetes, and CAD. However, there is no MR study to explore the causal relationship of GDF-15 level with major CVDs, such as stroke, AF, HF and myocardial infarction (MI).

Herein, we obtained genetic instruments and summary statistics for two-sample MR analysis from 5 GWASs to investigate the causal correlation between circulating GDF-15 levels and 9 CVDs, including any ischemic strokes (cardioembolic stroke, large-artery atherosclerotic stroke and small vessel stroke), AF, HF, nonischemic cardiomyopathy, CAD and MI. The present study concluded that GDF-15 might be a promising diagnostic biomarker and potential therapeutic target of some CVDs.

## Methods

### Instrument selection

Circulating GDF-15 level was predicted by the exposure genetically. A meta-analysis of GWAS which including 5440 individuals of European ancestry from four community-based cohorts (the mean age was 62 years and 53% were women) was utilized to obtain GDF-15 genetic associations [12], as described previously [13]. The selected single-nucleotide polymorphism (SNP) was associated with circulating GDF-15 levels at the genome-wide threshold (*p* < 5 × 10^–8^). All the SNPs were on chromosome 19 containing the PGPEP1 and MIC-1/GDF15 genes. LD-Link [14] was applied to test linkage disequilibrium between two loci in the same chromosome based on European ancestry. Every targeted SNP was searched in the PhenoScanner [15, 16] for the known effects of restricting the potential pleiotropy.

### Data for outcomes

The summary statistics for the selected SNPs with stroke were extracted from a large-scale meta-analysis of GWAS of 446,696 subjects of European ancestry (40,585 cases; 406,111 controls), conducted by the MESTROKE consortium [12]. Specifically, any ischemic stroke (AIS) cases were divided into three subtypes: cardioembolic stroke (CES), large-artery atherosclerotic stroke (LAS) and small vessel stroke (SVS). Genetic associations with AF were obtained from the largest meta-analysis of GWAS conducted by the AF consortium [17]. The study included 537,409 individuals of European ancestry. Summary statistics of HF come from 47,309 cases and 930,014 controls [18] and nonischemic cardiomyopathy (NICM) were extracted from a meta-analysis of GWAS of 488,010 European participants in the UK Biobank (1816 NICM cases) [19]. In addition, data of CAD and MI came from the meta-analysis of GWAS of 185,000 individuals [20].

### Statistical analyses

Two-sample MR method was performed to access the causality in genetic-predicted circulating GDF-15 levels with stroke, AF, HF, and NICM. The casual effect estimates of SNP instruments on CVD outcomes were calculated using the Wald Estimator [21], with standard error obtained using Delta method [22]. Then, odds ratios (OR) for each disease were meta-analyzed with the inverse-variance weighted (IVW) method to establish all SNPs valid or not [23]. Sensitivity analyses were conducted with both the Weighted Median and the MR-Egger method. The Weighted Median method allows half of the information comes from invalid instrumental variables [24]. The MR-Egger method not only detects pleiotropy with regression intercept but also all unbalanced directional pleiotropy [25]. Briefly, *p* values of < 0.05 were considered indicative of significant heterogeneity. All statistical analyses were performed by R version 3.6.1 (R Foundation for Statistical Computing, Vienna, Austria) and the MR package.

## Results

Among 9 SNPs which identified from the GWAS, 4 SNPs were discarded since rs1054564, rs3746181, rs1363120 were high linkage disequilibrium (LD) (r^2^ ≥ 0.8) and rs16982345 had not reach the genome-wide threshold (*p* > 5 × 10^−8^). The rest of SNPs (rs1227731, rs3195944, rs17725099, rs888663, rs749451) coming from chromosome 19 and containing the PGPEP1 and GDF-15 genes displayed in Additional file 1.

**Fig. 1:**
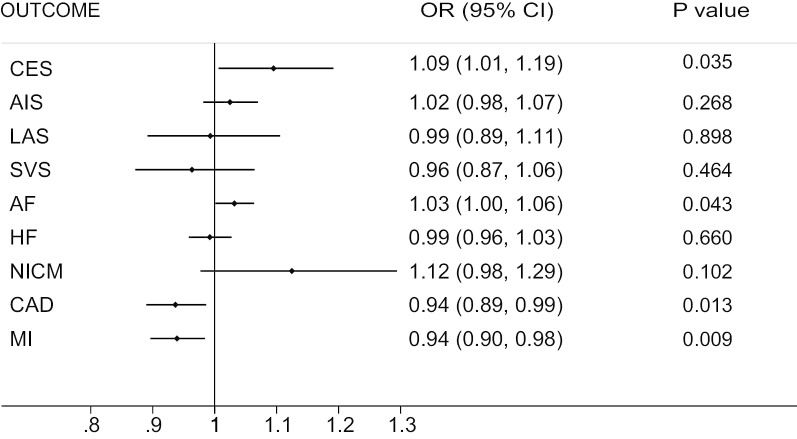
Forest plot of the relationship between GDF-15 and 9 CVDs. Data are reported as OR (odd ratio) and 95% CI ( confidence interval). *AIS* any ischemic stroke, *CES* cardioembolic stroke, *LAS* large-artery atherosclerotic stroke, *SVS* small vessel stroke, *AF* atrial fibrillation, *HF* heart failure, *NICM* nonischemic cardiomyopathy, *CAD* coronary artery disease, *MI* myocardial infarction

Casualty between circulating GDF-15 levels and 9 CVDs was shown in Fig. 1. For primary outcomes, significant causal association was observed between circulating GDF-15 levels with CES, AF, CAD and MI. Increasing of circulating GDF-15 level was significantly associated with the increased risk of CES and AF incidence (OR = 1.09, *p* = 0.035; OR = 1.03, *p* = 0.043, respectively) whereas reduced risk of CAD and MI incidence (OR = 0.94, *p* = 0.013; OR = 0.94, *p* = 0.009, respectively). While there was no robust evidence support the causal relationship of AIS, LAS, SVS, HF and NICM risk with circulating GDF-15 levels (*p* > 0.05). And validation and sensitivity analyses of relationship of circulating GDF-15 levels and outcomes conducted by IVW, MR-Egger test and Weighted Median method as shown in Table 1. No significant pleiotropic effects of the gene of GDF-15 were found in patients with either CES, AF, CAD, MI or other five CVDs.

**Table 1:** Results from two-sample MR analysis using 3 genetic instruments of GDF-15

Outcome	IVW			Weighted median			MR-Egger				
	Estimate	SE	*P* value	Estimate	SE	*P* value	Estimate	SE	*P* value	Intercept	*P* value
CES	0.091	0.043	0.035	0.111	0.053	0.036	0.154	0.150	0.304	− 0.017	0.660
AIS	0.024	0.022	0.268	0.022	0.027	0.409	0.104	0.077	0.175	− 0.021	0.279
LAS	− 0.007	0.055	0.898	− 0.013	0.062	0.829	− 0.033	0.189	0.862	0.007	0.886
SVS	− 0.037	0.051	0.464	− 0.037	0.057	0.523	0.030	0.177	0.86	− 0.018	0.689
AF	0.031	0.016	0.043	0.032	0.018	0.078	0.024	0.054	0.661	0.002	0.884
HF	− 0.008	0.018	0.660	0.012	0.023	0.589	0.002	0.072	0.973	− 0.003	0.881
NICM	0.117	0.072	0.102	0.106	0.084	0.204	0.207	0.249	0.406	− 0.024	0.708
CAD	− 0.065	0.026	0.013	− 0.062	0.029	0.032	− 0.010	0.101	0.921	− 0.015	0.569
MI	− 0.063	0.024	0.009	− 0.063	0.028	0.024	− 0.097	0.084	0.249	0.009	0.671

Data are reported as OR and 95% CI

*SE* Std error, *AIS* any ischemic stroke, *CES* cardioembolic stroke, *LAS* large-artery atherosclerotic stroke, *SVS* small vessel stroke, *AF* atrial fibrillation, *HF* heart failure, *NICM* nonischemic cardiomyopathy, *CAD* coronary artery disease, *MI* myocardial infarction

## Discussion

The present study investigated whether there was a causal association between circulating GDF-15 levels and nine CVDs including AIS, CES, LAS, SVS, AF, HF, NICM, CAD and MI by using two-sample MR based on 5 GWASs and 2.6 million cases. The result suggested that circulating GDF-15 level had a significant causal association with the incidence of CES, AF, CAD and MI, whereas no significant relationship could be concluded when coming to AIS, LAS, SVS, HF and NICM. In addition, a higher circulating GDF-15 was associated with the higher risk of CES and AF and lower risk of CAD and MI. Therefore, our study indicated a crucial role of circulating GDF-15 levels for the prevention and intervention of CVDs.

### GDF-15 and CES

CES has tripled in the past few decades and would persistently triple by 2050 worldwide. It’s the predominantly increasing proportion in ischemic strokes and eventually causes more severe strokes than other ischemic stroke subtypes [26]. The present result showed that patients with higher circulating levels of GDF-15 had more risk of CES. In ENGAGE AF-TIMI 48 trial, GDF-15 levels elevating from the baseline of 1661 pg/mL to 12 months of 1711 pg/mL were independently associated with a twofold higher rate of stroke or systemic embolic events in AF patients [27]. Likely, after 1.9 years of follow-up in ARISTOTLE trial, an annual rate of stroke or systemic embolic events was 2.03% in AF patients with the highest quartile of GDF-15 (> 2052 ng/L) compared to 0.90% in those with the lowest quartile of GDF-15 (≤ 977 ng/L) [28]. As AF is one of the most common risk factors for CES, some studies showed that GDF-15 could promote thrombosis in patient with AF by inhibiting anticoagulation. AF patients with high serum GDF-15 levels (range from 1661.0 to 5163.0 pg/mL) had 19.2% longer clot lysis time (CLT) (range from 94.3 to 117.5 min), indicating that elevation of serum GDF-15 levels drives anti-fibrinolytic reactions unrelated to increased plasminogen activator inhibitor-1 (PAI-1) or antiplasmin [29]. Taken together, circulating GDF-15 levels might exert a predictive value of CES in AF patients attributed to its prothrombotic effects.

### GDF-15 and AF

AF is the most common arrhythmia, currently affecting over 33 million individuals worldwide. The prevalence of AF is expected to more than double in adults over the next 40 years. AF is associated with a twofold increase in premature mortality and major adverse cardiovascular events such as HF, stroke and MI. Our result suggested that the circulating GDF-15 level was positive associated with the risk of AF. In community-based Individuals [30], postoperative patients [31] and hypertrophic cardiomyopathy (HCM) patients [32], those who had the higher GDF-15 levels were more vulnerable to AF than the lower. In addition, circulating GDF-15 level was reckoned independently associated with paroxysmal AF after multivariable analyses. In the previous study, the significantly higher serum levels of GDF-15 were found in patients with paroxysmal AF than those of controls (1473.14 ± 628.52 vs. 1233.592 ± 262.76 pg/ml, *p* < 0.05) [33]. Furthermore, serum GDF-15 level was associated with incident AF with the hazards ratios of 1.31 after adjusted for age and gender [30]. However, some inconsistent studies showed that serum GDF-15 level was not influenced by the presence of AF in patient with HF from the BIOSTAT-CHF trial [9] and patients with chronic kidney disease (CKD) from the CRIC study [34]. Potential pathologic mechanisms of AF include atrial remodeling, reentry, pulmonary vein trigger, abnormal autonomic nerve modulation and inflammation. Otherwise, atrial structural remodeling is closely related to atrial fibrosis and extracellular matrix. Some evidences showed that GDF-15 could enhance activation of M2 macrophages and induce fibroblasts to participate in the progression of cardiac fibrosis [35]. In addition, GDF-15 might be involved in the process of inflammatory and oxidative stress, which also could promote the occurrence of AF. Taken together, circulating GDF-15 level might be a potentially biomarker and therapeutic target for AF.

### GDF-15 and CAD

CAD is the most prevalent and important cause of ischemic heart disease, with major implications on global morbidity and mortality. The present result suggested that higher circulating level of GDF-15 was related to the lower risks of CAD and MI. GDF-15 may have a protective effect on atherosclerosis process. Consistently, overexpression of GDF-15 in macrophages significantly attenuated atherosclerotic lesions in the apoE knockout mouse model of atherosclerosis [36]. In addition, GDF-15 showed anti-apoptotic effect against ischemia reperfusion (I/R) and reduced the MI area mediated by activating intracellular phosphoinositide 3-kinase (PI3K)—protein kinase B (Akt)-dependent signaling [37]. Furthermore, GDF-15 could reduce platelet aggregation by inhibiting platelet integrin activation. GDF-15 knockout mice displayed an accelerated systemic thrombus formation and a reduced survival rate, while exogenous recombinant GDF-15 administration alleviated thrombus formation [38]. And the increased GDF-15 level may exert beneficial roles during process of CVD and MI as the FRISC-II trial suggested [39]. Taken together, GDF-15 might play multiple protective roles in the development and progression of CVD and MI.

### GDF-15 and HF

Though the present MR study did not support a causality of the circulating GDF-15 with the risk of HF, there are some reports that GDF-15 may be related to disease severity and prognosis HF patients [40]. In Val-HeFT trial, serum GDF-15 levels at baseline were abnormally high (> 1200 ng/L) in 85% of HF patients. Baseline serum GDF-15 levels (per 100 ng/L) were associated with the risks of mortality (hazard ratio 1.017; *p* < 0.001) and first morbid event (hazard ratio 1.020; *p* < 0.001). Increases in GDF-15 over 12 months were independently associated with the risks of future mortality and first morbid event. Taken together, serum GDF-15 concentration had not only a promising value of diagnosis but also a superior prognostic biomarker. However, patient with HF usually have some comorbidities such as hypertension, AF, stroke, diabetes, and chronic renal dysfunction, which may influence the circulating GDF-15 level and need to be eliminated.

### Strengths and limitations

This MR study firstly described the causal association between GDF-15 and 9 CVDs. Our work provided with a new perspective to clarify the role of GDF-15 in the development of CVDs. However, limitations should be considered while interpreting the results. The MR method have some common defects [41]. Firstly, the SNPs we selected could not satisfy the demand of independence principle. In addition, the MR is not sensitive to confounders from environmental exposures and might violate exclusion restriction unless we took into consideration all influence factors of GDF-15. Since our summary statistic was based on European populations, limiting the generalizability of our work, other high qualified databases should be enrolled to further strengthen the results.

## Conclusion

In summary, this MR study provides with genetic evidence for the causal relationship of circulating GDF-15 levels with the increased risk of CES, AF and reduced risk of CAD, MI, but not for AIS, LAS, SVS, HF and NICM. It indicates that GDF-15 might be a promising biomarker or potential therapeutic target for some CVDs.

## Supplementary information

**Additional file 1**. SNP predicting GDF-15 identified in GWAS.

## Data Availability

The datasets used and/or analysed during the current study are available from the corresponding author on reasonable request.

## Abbreviations

GDF-15: Growth differentiation factor 15
CVD: Cardiovascular disease
HFrEF: Heart failure with reduced ejection fraction
ACS: Acute coronary syndrome
CAD: Coronary artery disease
GWAS: Genome-wide association study
SNP: Single-nucleotide polymorphism
AIS: Any ischemic stroke
CES: Cardioembolic stroke
LAS: Large-artery atherosclerotic stroke
SVA: Small vessel stroke
AF: Atrial fibrillation
HF: Heart failure
NICM: Nonischemic cardiomyopathy
MI: Myocardial infarction

## References

[1] Fujita Y, Inagaki N (2017). Metformin: new preparations and nonglycemic benefits. Curr Diab Rep.

[2] Gerstein HC, Pare G, Hess S, Ford RJ, Sjaarda J, Raman K (2017). Growth differentiation factor 15 as a novel biomarker for metformin. Diabetes Care.

[3] Coll AP, Chen M, Taskar P, Rimmington D, Patel S, Tadross JA (2020). GDF15 mediates the effects of metformin on body weight and energy balance. Nature.

[4] Yang H, Filipovic Z, Brown D, Breit SN, Vassilev LT (2003). Macrophage inhibitory cytokine-1: a novel biomarker for p53 pathway activation. Mol Cancer Ther.

[5] Bouabdallaoui N, Claggett B, Zile MR, McMurray JJV, O'Meara E, Packer M (2018). Growth differentiation factor-15 is not modified by sacubitril/valsartan and is an independent marker of risk in patients with heart failure and reduced ejection fraction: the PARADIGM-HF trial. Eur J Heart Fail.

[6] Hagstrom E, James SK, Bertilsson M, Becker RC, Himmelmann A, Husted S (2016). Growth differentiation factor-15 level predicts major bleeding and cardiovascular events in patients with acute coronary syndromes: results from the PLATO study. Eur Heart J.

[7] Hagstrom E, Held C, Stewart RA, Aylward PE, Budaj A, Cannon CP (2017). Growth differentiation factor 15 predicts all-cause morbidity and mortality in stable coronary heart disease. Clin Chem.

[8] Wang TJ, Wollert KC, Larson MG, Coglianese E, McCabe EL, Cheng S (2012). Prognostic utility of novel biomarkers of cardiovascular stress: the Framingham Heart Study. Circulation.

[9] Santema BT, Chan MMY, Tromp J, Dokter M, van der Wal HH, Emmens JE (2019). The influence of atrial fibrillation on the levels of NT-proBNP versus GDF-15 in patients with heart failure. Clin Res Cardiol.

[10] Smith GD, Ebrahim S (2003). ‘Mendelian randomization’: can genetic epidemiology contribute to understanding environmental determinants of disease?. Int J Epidemiol.

[11] Cheung CL, Tan KCB, Au PCM, Li GHY, Cheung BMY (2019). Evaluation of GDF15 as a therapeutic target of cardiometabolic diseases in human: a Mendelian randomization study. EBioMedicine.

[12] Malik R, Chauhan G, Traylor M, Sargurupremraj M, Okada Y, Mishra A (2018). Multiancestry genome-wide association study of 520,000 subjects identifies 32 loci associated with stroke and stroke subtypes. Nat Genet.

[13] Au Yeung SL, Luo S, Schooling CM (2019). The impact of GDF-15, a biomarker for metformin, on the risk of coronary artery disease, breast and colorectal cancer, and type 2 diabetes and metabolic traits: a Mendelian randomisation study. Diabetologia.

[14] Machiela MJ, Chanock SJ (2015). LDlink: a web-based application for exploring population-specific haplotype structure and linking correlated alleles of possible functional variants. Bioinformatics.

[15] Staley JR, Blackshaw J, Kamat MA, Ellis S, Surendran P, Sun BB (2016). PhenoScanner: a database of human genotype-phenotype associations. Bioinformatics.

[16] Kamat MA, Blackshaw JA, Young R, Surendran P, Burgess S, Danesh J (2019). PhenoScanner V2: an expanded tool for searching human genotype-phenotype associations. Bioinformatics.

[17] Roselli C, Chaffin MD, Weng LC, Aeschbacher S, Ahlberg G, Albert CM (2018). Multi-ethnic genome-wide association study for atrial fibrillation. Nat Genet.

[18] Shah S, Henry A, Roselli C, Lin H, Sveinbjornsson G, Fatemifar G (2020). Genome-wide association and Mendelian randomisation analysis provide insights into the pathogenesis of heart failure. Nat Commun.

[19] Aragam KG, Chaffin M, Levinson RT, McDermott G, Choi SH, Shoemaker MB (2018). Phenotypic refinement of heart failure in a national biobank facilitates genetic discovery. Circulation.

[20] Nikpay M, Goel A, Won HH, Hall LM, Willenborg C, Kanoni S (2015). A comprehensive 1,000 genomes-based genome-wide association meta-analysis of coronary artery disease. Nat Genet.

[21] Lawlor DA, Harbord RM, Sterne JA, Timpson N, Davey Smith G (2008). Mendelian randomization: using genes as instruments for making causal inferences in epidemiology. Stat Med.

[22] Bautista LE, Smeeth L, Hingorani AD, Casas JP (2006). Estimation of bias in nongenetic observational studies using “Mendelian triangulation”. Ann Epidemiol.

[23] Burgess S, Bowden J, Fall T, Ingelsson E, Thompson SG (2017). Sensitivity analyses for robust causal inference from Mendelian randomization analyses with multiple genetic variants. Epidemiology.

[24] Bowden J, Davey Smith G, Haycock PC, Burgess S (2016). Consistent estimation in Mendelian randomization with some invalid instruments using a weighted median estimator. Genet Epidemiol.

[25] Burgess S, Thompson SG (2017). Erratum to: Interpreting findings from Mendelian randomization using the MR-Egger method. Eur J Epidemiol.

[26] Kamel H, Healey JS (2017). Cardioembolic stroke. Circ Res.

[27] Berg DD, Ruff CT, Jarolim P, Giugliano RP, Nordio F, Lanz HJ (2019). Performance of the ABC scores for assessing the risk of stroke or systemic embolism and bleeding in patients with atrial fibrillation in ENGAGE AF-TIMI 48. Circulation.

[28] Wallentin L, Hijazi Z, Andersson U, Alexander JH, De Caterina R, Hanna M (2014). Growth differentiation factor 15, a marker of oxidative stress and inflammation, for risk assessment in patients with atrial fibrillation: insights from the Apixaban for Reduction in Stroke and Other Thromboembolic Events in Atrial Fibrillation (ARISTOTLE) trial. Circulation.

[29] Matusik PT, Malecka B, Lelakowski J, Undas A (2020). Association of NT-proBNP and GDF-15 with markers of a prothrombotic state in patients with atrial fibrillation off anticoagulation. Clin Res Cardiol.

[30] Rienstra M, Yin X, Larson MG, Fontes JD, Magnani JW, McManus DD (2014). Relation between soluble ST2, growth differentiation factor-15, and high-sensitivity troponin I and incident atrial fibrillation. Am Heart J.

[31] Bening C, Mazalu EA, Yaqub J, Alhussini K, Glanowski M, Kottmann T (2020). Atrial contractility and fibrotic biomarkers are associated with atrial fibrillation after elective coronary artery bypass grafting. J Thorac Cardiovasc Surg.

[32] Montoro-Garcia S, Hernandez-Romero D, Jover E, Garcia-Honrubia A, Vilchez JA, Casas T (2012). Growth differentiation factor-15, a novel biomarker related with disease severity in patients with hypertrophic cardiomyopathy. Eur J Intern Med.

[33] Shao Q, Liu H, Ng CY, Xu G, Liu E, Li G (2014). Circulating serum levels of growth differentiation factor-15 and neuregulin-1 in patients with paroxysmal non-valvular atrial fibrillation. Int J Cardiol.

[34] Lamprea-Montealegre JA, Zelnick LR, Shlipak MG, Floyd JS, Anderson AH, He J (2019). Cardiac biomarkers and risk of atrial fibrillation in chronic kidney disease: the CRIC study. J Am Heart Assoc.

[35] Takenouchi Y, Kitakaze K, Tsuboi K, Okamoto Y (2020). Growth differentiation factor 15 facilitates lung fibrosis by activating macrophages and fibroblasts. Exp Cell Res.

[36] Johnen H, Kuffner T, Brown DA, Wu BJ, Stocker R, Breit SN (2012). Increased expression of the TGF-b superfamily cytokine MIC-1/GDF15 protects ApoE(-/-) mice from the development of atherosclerosis. Cardiovasc Pathol.

[37] Kempf T, Eden M, Strelau J, Naguib M, Willenbockel C, Tongers J (2006). The transforming growth factor-beta superfamily member growth-differentiation factor-15 protects the heart from ischemia/reperfusion injury. Circ Res.

[38] Rossaint J, Vestweber D, Zarbock A (2013). GDF-15 prevents platelet integrin activation and thrombus formation. J Thromb Haemost.

[39] Wollert KC, Kempf T, Lagerqvist B, Lindahl B, Olofsson S, Allhoff T (2007). Growth differentiation factor 15 for risk stratification and selection of an invasive treatment strategy in non ST-elevation acute coronary syndrome. Circulation.

[40] Anand IS, Kempf T, Rector TS, Tapken H, Allhoff T, Jantzen F (2010). Serial measurement of growth-differentiation factor-15 in heart failure: relation to disease severity and prognosis in the Valsartan Heart Failure Trial. Circulation.

[41] Smith GD, Ebrahim S (2004). Mendelian randomization: prospects, potentials, and limitations. Int J Epidemiol.

