# Supplementary material for: The impact of growth differentiation factor 15 on the risk of cardiovascular diseases: two-sample Mendelian randomization study
Source: BMC Cardiovasc Disord. 2020 Oct 28;20:462. doi: 10.1186/s12872-020-01744-2 (PMC7594331; doi:10.1186/s12872-020-01744-2)
Supplement: Supplementary file 1 — Additional file 1. SNP predicting GDF-15 identified in GWAS. [file 12872_2020_1744_MOESM1_ESM.pdf]

Additional file 1: SNP predicting GDF-15 identified in GWAS

SNP	Gene	Effect allele	Other allele	Beta Standard	error	P value
rs1227731	GDF15	A	G	0.3085	0.0257	3.37E-33
rs3195944	PGPEP1	G	A	0.3344	0.0292	2.39E-30
rs17725099	PGPEP1	A	G	0.1346	0.0245	4.13E-08
rs749451	PGPEP1	C	T	0.2178	0.0187	2.54E-31
rs888663	PGPEP1	T	G	0.3029	0.0244	2.64E-35
